# Wool-Reinforced Cement Based Composites

**DOI:** 10.3390/ma13163590

**Published:** 2020-08-14

**Authors:** Daria Jóźwiak-Niedźwiedzka, Alessandro P. Fantilli

**Affiliations:** 1 Department of Experimental Mechanics, Institute of Fundamental Technological Research, Polish Academy of Sciences, Pawińskiego 5B, 02-106 Warsaw, Poland; djozwiak@ippt.pan.pl; 2 Department of Structural Engineering, Construction and Soil Mechanics, Politecnico di Torino, Corso Duca Degli Abruzzi 24, 10129 Torino, Italy

**Keywords:** natural fibres, sheep wool fibres, mechanical properties, durability, microstructure

## Abstract

In this paper, an overview of the latest research activities in the field of cement-based composites incorporating sheep wool reinforcement is presented. First, the characteristics of this type of natural fibre are described. Then, the current use of sheep wool fibres in cement-based composites is discussed. The research problems regarding the properties of cement matrix composites reinforced with sheep wool are divided into four groups: thermal and acoustic properties, mechanical behavior, durability issues, and microstructure aspects. The latter two groups are analysed separately, because both durability and microstructure are of particular importance for future applications of wool reinforcement. Finally, the main directions of future researches are presented.

## 1. Introduction

The general purpose of fibres application in cement-based composites is to increase material toughness by improving the resistance to crack propagation. The reinforcement also increases the composite material tensile strength, especially when a large quantity of fibres is added to the cementitious matrix [1]. Various kinds of dispersed reinforcement in the form of thin fibres are used in concrete structures. Depending on the type of material, the fibres can be distinguished as metallic and non-metallic: glass, basalt, natural organic, and polymeric (like polyethylene, nylon, polyester, Kevlar, PVA-poly(vinyl alcohol).

The use of both metallic and non-metallic fibres to improve concrete behaviour in tension is not new. However, in recent years there has been growing interest in utilizing natural fibres (from plants and animals) to produce eco-friendly construction materials. The relatively high cost of industrial fibres and the aim of reducing the negative environmental impact of the construction industry make the use of natural fibres more common [2]. The availability of natural fibres is directly related to the climatic zones. This is particularly true for plant fibres, like jute [3], coir [4], sisal [5,6], bamboo [7], wood [8], palm leaf [9], coconut leaf [10] and fibres [11], cotton [12] and hemp [13], or cellulose [14]. Plant or cellulose fibres have many advantages, such as wide availability at a relatively low cost, biological renewal, recyclability, biodegradability, harmless nature, and zero carbon footprint [15]. However, the same properties can also be attributed to animal origin fibres, but with better mechanical properties, especially in the case of wool [16].

Several studies on the use of sheep’s wool in the construction industry are related to the applications as an insulating material, both thermal and acoustic [17]. Indeed, sheep wool is comparable to other insulation materials, such as mineral wool and calcium silicate [18]. The results of some experimental measurements show that sheep wool is competitive in terms of thermal conductivity and acoustic absorption [19,20,21,22,23,24,25]. Korjenic et al. [18] showed that sheep wool is characterized by high hygroscopicity, which made it capable to absorb moisture, prevent condensation and regulate humidity in insulation materials. Due to the high content of water and nitrogen, wool is also a naturally flame retardant [26]. Sheep’s wool is also an excellent acoustic material but, according to Zach et al. [27], no additional acoustic benefits are achieved with material thicknesses greater than 170 mm.

An undoubted advantage of sheep wool is the influence with human health. Unlike fibre glass, wool can be installed without protective clothing, because it does not cause irritation to the skin, eyes or respiratory tract [20]. The research conducted by Liang and Ho [28] revealed that the toxicity of combusted insulating materials, such as rock wool and fibreglass, is significantly higher than that of organic materials. Wool can also absorb unhealthy carbons in the atmosphere, helping to provide a cleaner environment [26].

Despite all the above-mentioned benefits, the large-scale production of cement-based composites reinforced with sheep wool fibres is currently limited by the long-term durability [29]. However, in all the previous researches, including the recent review papers by Parlato and Porto [30] and Allafi et al. [31], there is no information on the durability of cement-based composites with the addition of wool fibres.

The durability issue is associated with an influence of the pH of cement matrix on the sheep wool fibres. Research conducted by Fantilli and Jóźwiak-Niedźwiedzka [29] showed direct effect of cement alkalinity and curing conditions on durability of wool fibres in cement-based mortars. The capability of the sheep wool fibres to bridge the crack surfaces, and to guarantee the presence of a residual tensile strength in the post-cracking stage is remarkably reduced in high alkali cement and in high humidity conditions. This phenomenon is particularly highlighted by the values of the residual strength and the fracture toughness in bending of wool-reinforced cement-based mortars.

To mitigate the degradation of wool fibres in cement-based composites, two main methods have been adopted: fibre pre-treatment or cement matrix modification. Attempts have been made to modify the surface of the fibres to improve their mechanical properties and durability [30,32,33], and to reduce concrete’s alkalinity by partially replacing the cement with supplementary cementitious materials [34], or use blended cement [35] and/or low-alkali cement [29].

Reducing the clinker content in the cementitious matrix through the use of supplementary cementitious materials improves the durability of natural fibres by increasing cement hydration, and reducing the alkalinity of pore solutions and Portlandite consumption [36]. The modification of cement hydration may require less effort and involve less cost compared to pre-treatment of natural fibre. In addition, the possible influence of the modifying agent on the cement matrix should also be considered [36].

All the above-mentioned researches concerning sheep wool fibres application in the cement-based composites are reviewed and presented in this paper. Specifically, the main material properties, the applications in building materials, and the behaviour of cement-based matrix composites reinforced with sheep wool fibres are described.

## 2. Characteristics of Sheep Wool Fibres

**Figure 1:**
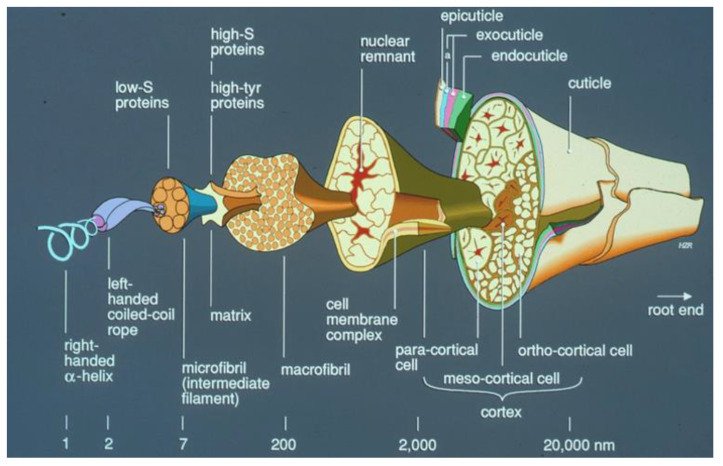
Schematic diagram of wool fibre structure [37] (2020, https://www.hdwool.com/blog/the-structure-of-wool).

Wool is the natural protein fibre deriving from the fleece of sheep. It has one of the most complex structure among textile fibres (Figure 1), as a single wool fibre consists of a cortex and a surrounding cuticle layer.

Each of the two components is formed of various other morphological components. The cortex contains cortical cells and the cell membrane complex [26]. It has a bilateral structure and is responsible for the mechanical behaviour. Cortex is the carrier of the characteristics of wool properties such as elasticity, ductility, and swelling force [21].

**Figure 2:**
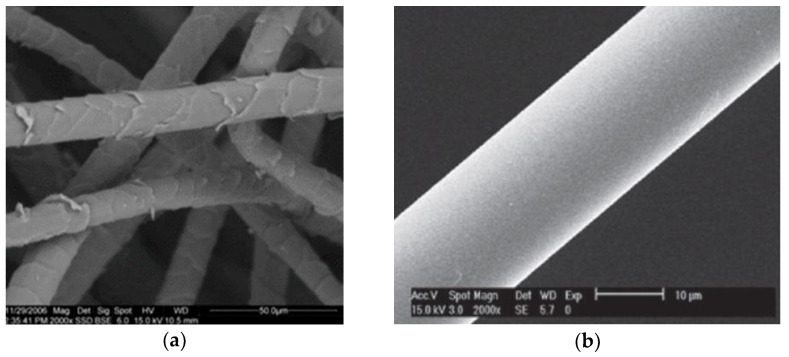
SEM image of (**a**) sheep’s wool fibre showing its surface scales [30] (CC BY 4.0, 2020, Sustainability), and (**b**) man-made synthetic smooth glass fibre [38] (BY-NC-ND 3.0, 2017, E-Polymers).

Wool fibres have a particular surface structure of overlapping scales called cuticle cells, which anchor a fibre in the sheep’s skin. The surface of wool fibres (Figure 2a) is very different with respect to typical man-made fibres, which have a very smooth surface (Figure 2b) [2].

Wool fibre has similar properties whatever the origin of the sheep [26]. Regardless of the origin, wool shows similar percentages of carbon (~50 wt.%), hydrogen (~7 wt.%), oxygen (~22 wt.%), nitrogen (~16 wt.%), and sulphur (~5 wt.%). The high sulphur content in wool comes mainly from the high cystine content of these fibres. Trace elements can also be detected, such as Ca, Cd, Cr, Cu, Hg, Zn, Pb, Fe, As, and Si, which are incorporated into keratin from extraneous sources [26].

The properties of wool depend on its chemical composition, and on the complex protein structure. In the natural state, raw sheep wool contains a number of constituents other than the fibre, such grease, water-soluble material derived from perspiration and contaminants (i.e., feces and vegetable matter) which can be easily removed [39]. Regardless of the final application, washing in hot soapy water is the first step in the wool cleaning process to remove dirt and grease. The scouring water (at 65 °C) does not dissolve the wax (i.e., lanolin), and a detergent is added to remove the dirt and to emulsify the wax. In this stage, also other chemical agents are applied to the wool on the basis of the final use [2,39].

Clean wool contains 82% of the keratinous proteins, which in turn contain high concentration of sulphur (~5%). The amount of sulphur in the keratin creates strong disulphide bonds and determines the strength of wool. Moreover, keratin does not dissolve in cold or hot water and does not breakdown into soluble substances [39].

Wool is characterized by a tensile strength of 120~180 MPa, an elongation at break of 25~35% and of a Young Modulus of 2.3–3.4 GPa [16,17]. A typical stress–strain curve of wool fibre, measured under tensile actions at 20 °C with 65% of relative humidity conditions is characterized by three regions, which in turn are differently affected by humidity. After decrimping, in the first region stress increases linearly up to a strain of 1~2%. After this point, elongation increases rapidly compared to small increases in stress. This section of the curve is known as the yield region, which ends at around 25~30% of elongation. Finally, the post-yield region shows a strain hardening up to the rupture of the fibre. The three slopes of the initial, yield and post-yield regions are in the approximate ratio of 100:1:10, respectively [26].

The humidity content significantly affects the stress–strain diagram of sheep wool fibres. The higher the moisture content, the further the fibre can be stretched. Dry fibre breaks at about 30% of elongation, whereas a wet fibre (100% humidity) does not break until an elongation of 70%. After stretching, fibre returns rapidly and completely to the original length [21]. If the wool fibre is extended by only 30% of its length, or less, and if the load is then removed and the fibre immersed without tension in water for 24 h, the fibre returns to its original length; and if the load-extension curve of the fibre is determined a second time, it follows the path of the first load–extension curve. These recovery properties are unique, and no other natural or synthetic fibre shows similar behaviour [40].

**Figure 3:**
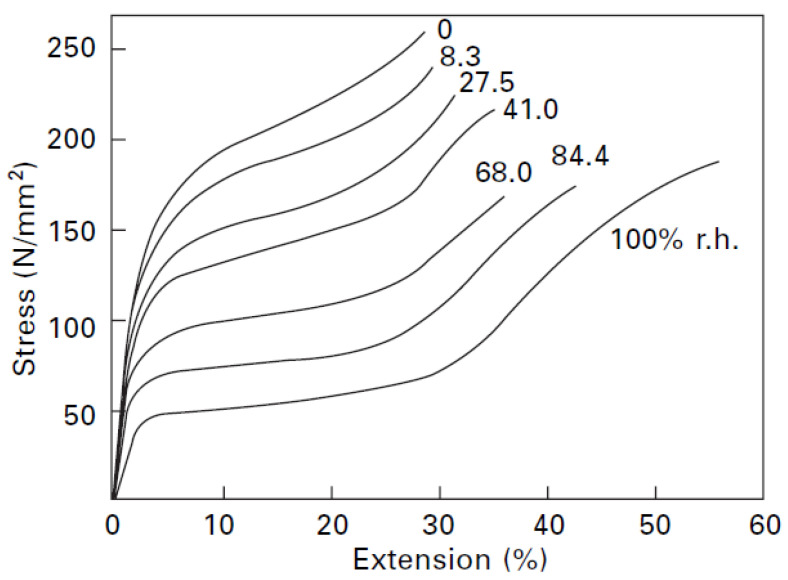
Effect of relative humidity on stress–strain curves of wool at room temperature [41] (RightsLink, License numer 4875281426526, 2008, Physical properties of textile fibres, 4th ed.).

In Figure 3, the influence of the relative humidity on the stress–strain curves of wool at room temperature is presented.

The major visible effect is the increment of the yield point. The mechanical properties of fibres also change with temperature. The differences between the behaviour of wet fibres at 20 °C and 95 °C revealed that the tenacity and stiffness were lower at the higher temperature, but the breaking extension was higher. Prolonged exposure to high temperatures can lead to the permanent degradation of fibres [41].

Wool fibres are insoluble in almost all solvents, except for alkalis, which damage them even in diluted solutions. Generally, it is accepted that fibres are damaged when pH is above 11, and the increment of temperature also accelerates the dissolution reaction [21]. Both the effects of acid and alkali cause a decrease in the strength of the wool fibre [42]. The strength of wet wool depends, to a large extent, on the covalent cross-links on the disulphide bonds [42].

## 3. Application of Sheep Wool in Cement-Based Composites

The sheep wool is an eco-friendly material, annually renewable, and totally recyclable which meets the requirements of green building components and therefore it is increasingly used in the building materials technology [30]. Also, the wool fibres are non-flammable. They require more oxygen to burn than is available in the air, making them a superior fibre for fire safety. Furthermore, they do not melt, drip or stick to the skin when they burn [26]. It does not burn and does not contribute to the propagation of flame, but carbonizes itself. This phenomenon is associated with its high content of nitrogen, which does not support combustion [43].

Among others, the above properties of sheep wool make them a good building insulation material with the desired thermo-hygrometric and acoustic properties. The sheep wool insulation materials which are already available for building technology can be divided into two categories [19]:

- Soft mats made of sheep wool, with thickness of 50 ± 10 mm, mainly used for the insulation of pitched roofs.
- Semi-rigid panels made of sheep wool fibres (75 ± 5%) and polyester fibres (25 ± 5%), with thickness 85 ± 35 mm.

The detailed information about thermal and acoustic properties of cement-based composites reinforced with sheep wool are presented in Section 4.1.

**Figure 4:**
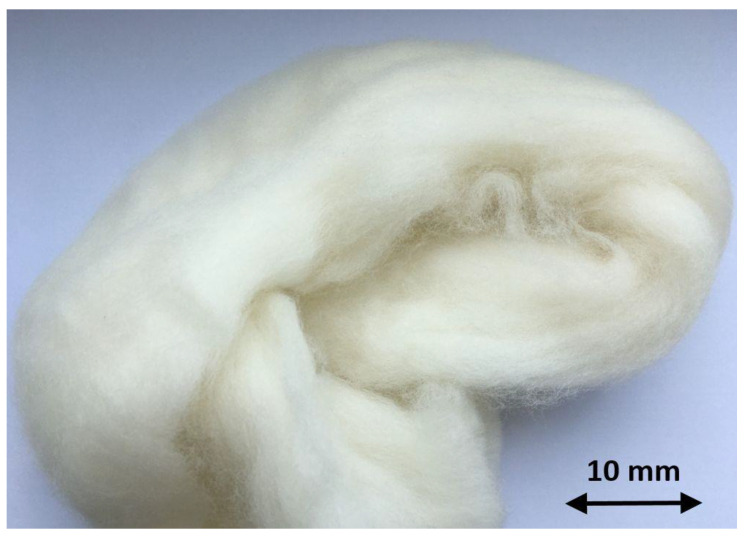
Sheep wool used in cement based composites.

Another application of sheep wool in cement-based composites is fibre reinforcement. Fibrous material was used in cement matrix composites in both mortar [29,44] and concrete [33]. Fantilli and Jóźwiak-Niedźwiedzka [29,44] analysed the application of sheep wool (Figure 4) as mortar reinforcement.

They revealed that the alkalinity of the cement strongly influenced the resistance of wool fibres dispersed in cementitious matrix. The lower the alkalinity of the cement paste, the better the resistance of wool fibres, which guaranteed larger post-cracking residual stresses in the wool-reinforced mortars. Regarding the performance of wool fibres as concrete reinforcement, they are comparable or slightly inferior to polypropylene fibres [43]. However, finding a way to improve the workability of concrete reinforced with wool fibres can significantly improve the fresh and hardened properties of sheep wool fibre reinforced concrete. Alyousef et al. [45] recommended to use the HRWR (high range water reduced admixture) to achieve the proper workability of fresh concrete mix, which decrease with the content of fibres. They also showed that all analysed concretes containing sheep wool fibres were characterized by higher tensile and flexural strength values than those of plain concrete. More information about mechanical properties of cement-based composites reinforced with sheep wool are presented in Section 4.2.

Dénes et al. [43] stated that wool can be used as carbon fibre precursor. Preliminary research showed that wool fibres can replace the synthetic polymer in the sight of carbon fibre production. Hassan et al. [46] found that carbon fibres were able to be produced through the carbonization of untreated and crosslinked wool fibre. The carbon yield of the resulting fibres was found to be a function of the type of crosslinking agents applied to wool. In addition, due to the importance of using locally available materials for rural building renovation as well as for restoration and repair of historic and cultural heritage buildings, the use of sheep wool is strongly suggested [18]. In fact, wool reinforced composites are suitable for the renovation of traditional buildings due to the comparable composition of the mixture with the original mortars [39].

## 4. Properties of Cement-Based Composites Reinforced with Sheep Wool

### 4.1. Thermal and Acoustic Properties

Sheep wool is regarded as one of the most performative insulating natural materials due to its thermo-hygrometric and acoustic properties [22,30,43,47,48]. One of the most important factors concerning the thermal insulation is thermal conductivity of material. To be considered as an insulation material, thermal conductivity should be less than 0.065 W/mK, *λ* [43]. As this value varies between 0.033 and 0.063 W/mK in the case of wool [21,23,43], it can be considered as a good insulation material. The research conducted by Korjenic et al. [ 18] showed that sheep wool, compared with mineral wool and calcium silicate, provides comparable thermal insulation characteristics, and in some applications even reveals better performance. Comparing the properties of sheep, flax and glass wool, Tuzcu [21] found that *λ* was 0.033, 0.040, and 0.034 w/mK, whereas specific heat capacity *c* was 1720, 1550, and 799 J/kgK, respectively. However, the thermal conductivity varies depending on the humidity conditions: *λ* increases with the content of water in the sheep wool or with the increment of the apparent density [22]. Volf et al. [23] investigated the treated sheep wool and raw sheep wool as natural insulating materials. They revealed that both types of wool had the lowest value of volumetric heat capacity *c_p_* (0.05 and 0.06 Jm^−3^K^−1^, respectively) and the highest value of thermal conductivity *λ* (0.063 and 0.062 Wm^−1^K^−1^) compared to mineral wool (*c_p_* = 0.09 Jm^−3^K^−1^ and *λ* = 0.039 Wm^−1^K^−1^) as well as to flax, hemp, and wood fibres. They concluded that natural insulations had comparable thermal properties to common building insulation materials and could bring advantages in thermal and moisture buffering.

Some researchers showed that, with the addition of sheep wool, density and thermal insulation improve, but, at the same time, the mechanical properties of the composite decrease [39]. Fiore et al. [24] investigated the mechanical behavior and thermal conductivity of a cement mortar with various length and different contents (i.e., 13%, 23%, and 46% by wt. of cement) of wool fibres. They revealed that the application of wool fibres improved the thermal insulation in the analysed cement-based composites. 

Sheep wool shows good acoustical performances by absorbing and reducing noise [18]. According to Asdrubali [25] panels made from sheep wool were characterized by an absorption coefficient *α* of about 0.84 at 2000 Hz, slightly lower than rock wool (0.91) or polyester (0.95), but significantly higher than cellulose (0.53) or hemp fibres (0.52). A sheep wool panel of 20 mm thickness had also a very low index of impact noise reduction Δ*L_w_* (18 dB), much smaller than glass wool (31 dB) or expanded polystyrene (30 dB), even lower than wood wool (21 dB) or cellulose (22 dB). 

Wool fibres are more hygroscopic than any other fibres. As a result, when moisture content increases, the thermal conductivity coefficient does not change significantly [48].

### 4.2. Mechanical Behavior

The use of natural fibres as a reinforcement of cement-based composites can increase the toughness of concretes and mortars, and represents a sustainable option to the traditional industrial fibres as well. Indeed, such fibres can bridge the surfaces of the cracks in the post-cracking stages and reduce the environmental impact of the construction industry [32]. 

Porubská et al. [49] investigated the gamma radiation up to 400 kGy on the mechanical properties of sheep wool. They found that the tensile strength at failure did not change significantly while the original elongation firstly increased and, then, a monotonous reduction was observed. Grădinaru et al. [50] examined the influence of sheep wool fibres and fly ash on the compressive and tensile strength of concrete. They tested seven types of mixtures, with and without the addition of fly ash, and of two percentages (i.e., 0.35% and 0.80% in weight) of wool fibres (with a length comprised by 25 mm and 55 mm). The experimental results showed that sheep wool fibres did not improve the strength of the concrete at the studied percentages of addition and, in most of the cases, a lower strength was measured. It was observed that when sheep wool fibres are used, compressive strength reduced of 15–30%, compared to the reference concrete, although the degree of reduction depended on the fibre length and dosage. A fibre length of 55 ± 5 mm and a dosage of 0.35% had an insignificant influence on the compressive strength, but a higher dosage or a smaller length of the fibre decreased the value of the compressive strength. Fiore et al. [24] investigated the mechanical behaviour of a cement mortar with various length and different contents (i.e., 13%, 23%, and 46% by wt. of cement) of wool fibres. They revealed that wool-reinforced composites showed lower compressive strength than the reference no wool cement composite, regardless of the content and length of fibres. Similar conclusions were presented by Cardinale et al. [51]. In their research, the addition of sheep wool fibres was much smaller, 2%, 5%, and 7% per dry raw materials mass. They investigated flexural and compressive strength of mortars made with CEM II/A RCK 42.5 N, crushed sand of 0.63 mm, lime and water. As a result, a reduction of flexural and compressive strength of 9.1% and 14.7% was respectively observed for mortar with 2% of wool fibres. In mortars with a higher content of wool, the decrease of strength was much greater (more than 80%).

Opposite results were obtained by Fantilli et al. [32]. They analysed the influence of sheep wool fibres on the mechanical properties of cementitious mortars. Additionally, mortars reinforced with hemp were also tested. The authors stated that the flexural strength and the ductility increased when wool is added to cementitious mortars. Similar to other natural fibres, wool improved the mechanical and ecological performances of the mortars. Pederneiras et al. came to the same conclusions [52]: the use of wool fibres in cement mortars improved the flexural strength. A higher increase in flexural strength was observed for longer fibres (30 mm) in comparison to shorter fibres (15 mm). The cement-based mortar made with CEM II/B-L 32.5 N and 20% of 30 mm long wool fibres revealed an increase of 40% and 26% in flexural and compressive strength, respectively. Alyousef et al. [45] revealed that sheep wool fibres (up to 1.5% of 70 mm length fibres) can reduce the compressive strength of concrete, but undoubtedly improve the tensile and flexural strength, and concrete ductility (with higher energy absorption capacity) as well. 

Sheep wool fibres as reinforcement in lime based composite materials were investigated by Tămaş-Gavrea et al. [53]. They analysed a mortar containing hydrated lime, rice paste and sheep wool fibres, and stated that this composite was characterize by acceptable adhesive strength (equal to 0.125 N/mm^2^). 

Wool, kenaf, and wheat straw used as fibres, and clay used as a binder, were analysed in some studies conducted by Erkmen et al. [54]. Among the results, insulating materials containing 7% of wool fibres revealed the best result concerning compressive strength (4.9 MPa), thermal conductivity coefficient (0.061 W/mK), and water absorption (% 0.0015/h) in comparison to the other commercial products.

### 4.3. Durability and Microstructure

The durability of wool reinforced cement-based composites depends on the conditions of exploitation and on external actions. The bonding between fibres and the cementitious matrix is a decisive element. The latter depends on the quality and processes that appear in the fibre/matrix interface. It has been shown that in glass fibre-reinforced cement-based composites, the chemical interaction between these two constituents may be destructive for the composite integrity [1]. The usefulness of natural fibres in cement-based materials is limited by their high potential to degrade in alkaline environment. Frequently, they loss the strength when used as reinforcement of a cementitious matrix exposed to aggressive environmental conditions [29,35,36,55]. 

As it was expected, the addition of sheep wool fibres significantly influences the workability of fresh mixes. Cardinale et al. [51] tailored some cement-based mortars with a constant water/cement ratio (equal to 0.4) and various content of sheep wool fibres. They found deteriorated mortars due to the insertion of ever-increasing percentage of wool fibres, and the necessity of increasing the programmed quantity of water, in order to ensure the workability of the mixture. Similarly, Alyousef et al. [45] investigated the properties of fresh concrete (made with Ordinary Portland cement, natural aggregate, w/c = 0.5, and up to 6% of sheep wool fibres by weight of cement), and stated that addition of sheep wool fibres caused a huge demand of water for making the concrete workable. The reason of such low workability has to be ascribed to the high specific surface area and fineness of wool fibres. Thus, workability of concrete containing sheep wool fibres decreases with the increasing content of wool. If for reference concrete the slump value is 30 mm, for the same concrete with 2% of sheep wool fibres it decreased to 8 mm. Even the pretreatment of fibre with salty water, used to increase the surface friction, did not improve fresh concrete properties [33]. The slump value was 55 mm for reference concrete without fibres, and 22 mm for 2% of both unmodified and modified sheep wool fibers. Obviously, this negative phenomenon could be minimized by the addition of chemical admixtures.

**Figure 5:**
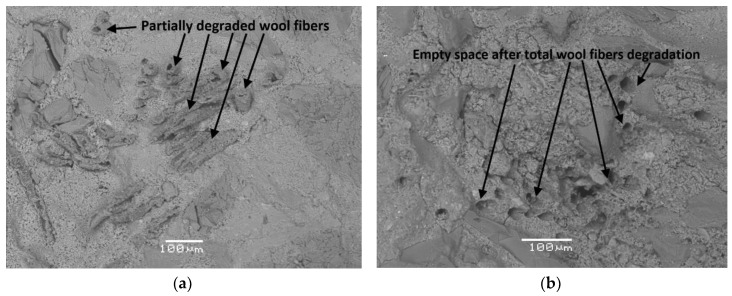
The microstructure of the mortar samples made with high-alkali cement and wool fibres addition cured at 20 °C: (**a**) 3 days and (**b**) 27 days, scale bar = 100 µm.

The degradation mechanisms of natural fibre in the alkaline and mineral-rich environment, which is typical of the cement-based matrixes, was investigated by Wei and Meyer [36]. They studied the degradation mechanisms and found that, by reducing alkalinity of pore solution, metakaolin effectively mitigates the deterioration of natural fibre. Also, the alkali degradation process of natural fibre was proposed. Cement hydration was presented to be a crucial factor in understanding fibre degradation behaviour, which is confirmed by the test results conducted by Fantilli and Jóźwiak-Niedźwiedzka [29]. They analysed the influence of the alkalinity of Portland cement type I and curing conditions on the mechanical properties and microstructure of sheep wool reinforced mortars. The results revealed that the lower the alkalinity of the cement paste, the better the resistance of wool fibres in cementitious matrix, which increased the residual stress after cracking in wool reinforced mortars. The curing of mortar beams in water at room temperature significantly accelerated the process of wool fibre degradation in matrix made with high-alkali cement (Na_2_O_eq_ = 1.1%), compared to those obtained with normal- and low-alkali cement (Na_2_O_eq_ = 0.6 and 0.4%, respectively). In Figure 5, the microstructure of the specimens made with high-alkali cement and wool fibres, after 3 and 27 days of curing in water at 20 °C, are presented.

It can be observed that, with high-alkali cement, the longer the time of curing in water, the higher the degree of wool fibres degradation.

**Figure 6:**
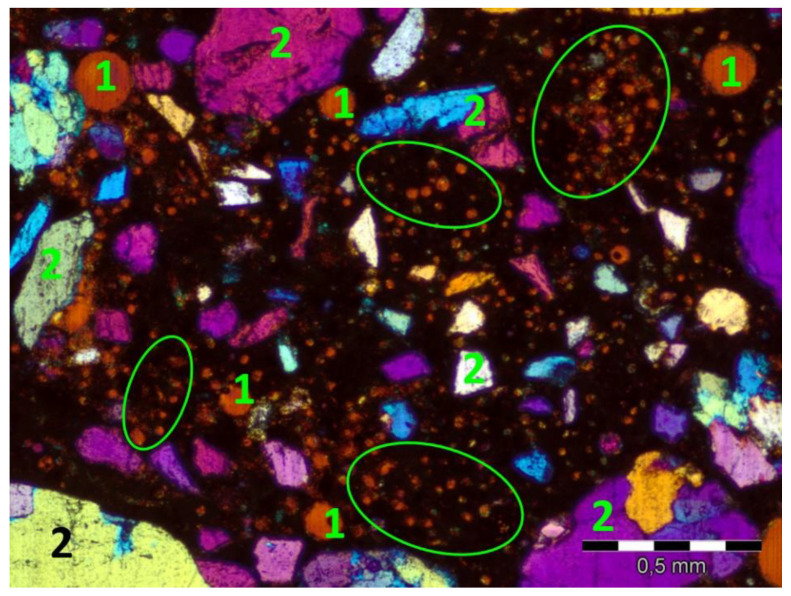
Thin section photograph of the microstructure of sheep wool reinforced mortar cured in water for 27 days in 20 °C, plane polarized light with gypsum plate, areas with visible wool fibre cross sections are marked, 1—air-voids, 2—fine aggregates, scale bar = 0.5 mm.

Research conducted by Fantilli et al. [35] on the compatibility between wool and polypropylene fibres and cement-based matrix (made with CEM II/B-LL 32.5 R) showed the influence of curing condition on durability of fibres. The beneficial effect of wool was not observed when the specimens were stored in water at 20 °C for 27 days (see Figure 6). Nevertheless, wool filaments were able to resist more than three days in the alkaline environment before their complete dissolution.

Thus, they can be used to contrast the effects of plastic shrinkage, as the industrial polypropylene fibres do.

The method of pre-treatment of sheep wool fibres are not always effective. Alyousef et al. [33] used saltwater treatment modification of sheep wool which caused an improvement of the fibre’s mechanical properties and improved adhesion with cement paste. Also, atmospheric plasma was used to modify the nano-metric properties of the fibre surface [32,56]. Ceria et al. [56] analysed the influence of the atmospheric plasma jet treatment on physical and mechanical properties of wool fabrics. Their researches revealed the increment of tensile strength (up to +13%) and elongation at break (up to +19%) by increasing the intensity of the plasma treatment. Hence, Fantilli et al. [32] treated sheep wool fibres with atmospheric plasma in order to modify the nanometric properties of their surface. However, a significant effect of treated wool fibre surface modification on the mechanical properties of cement-based mortars was not observed. Conversely, they found that, both the flexural strength and the ductility increased when wool, treated or not, was added to cementitious mortars.

**Figure 7:**
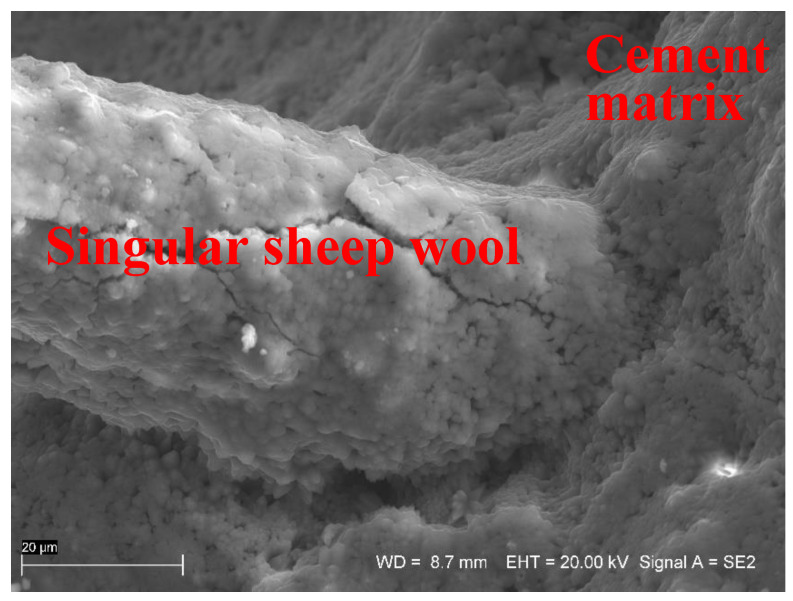
SE image of the sheep wool fibre—interface—cement matrix, scale bar = 20 µm.

As interface plays an important role in long-term durability of fibre reinforced cement-based composites, its characterization is important for service life modelling and prediction. Interfaces of fibre reinforced cement-based materials are quite porous with low strength and stiffness. The durability of materials such as natural fibres is strongly associated with an increase bond strength and loss of the flexibility of fibre bundles [57]. Savastano et al. [58] investigated the microstructure of cement-based materials containing natural fibres, namely sisal and banana pulp. Sisal fibres showed satisfactory bonding to the cement matrix, contrary to banana pulp. In all analysed composites, partial fibre debonding and matrix micro-cracking were dominant at the interfaces. However, the evidence of a porous transition zone or massive concentration of calcium hydroxide at the interface was not found. For 250 days of curing, a high porosity was not detected in the interfacial area and just one EDS spot indicated the presence of calcium hydroxide close to the fibres. The above conclusion is consistent with observation regarding the interface between sheep wool fibre and cement matrix (see Figure 7).

The SEM image of Figure 7 shows a very concise and homogeneous bond between the cementitious matrix and sheep wool fibre. The fibre covering of hydration products is also quite well visible, which may suggest a good fibre–matrix adhesion. A denser matrix in the fibre-matrix transition zone can lead to higher bond, resulting in higher strength, lower toughness and a greater probability of fibre failure by fracture rather than by pullout. Microcracks, which are visible on the wool fibre surface (Figure 7), formed during the bending test on mortar beams.

There is no available information about durability of wool-reinforced cement-based composites as a function of permeability. Based on the results of the researches conducted by Giosué et al. [59] and Zhao et al. [60], cautions should be taken when the wool-reinforced composites have to be use in humid environment, or the mortar contains chloride ions, such as those of the marine environment. Giosué et al. [59] noted an increase of 30% in total open porosity of hydraulic lime-mortars containing 25% of wool fibre, when compared to the reference mortar without fibres. Similarly, Zhao et al. [60] investigated the natural plant fibre-reinforced cement-based composites containing pineapple leaf fibre and ramie fibre. They found that the coefficient of capillary absorption and chloride diffusion of tested composites were significantly larger than the plain composites, and the difference was evident with the increment of fibre volume fraction.

## 5. Conclusions

Wool is one of the earth’s most sustainable resources due to its natural, renewable, sustainable, biodegradable, low carbon impact, energy efficient properties. Several researches revealed the very good thermal and acoustic properties of cement-based composites, like soft mats, panels, and facades, reinforced with sheep wool. A special treatment of sheep wool fibres as well as low alkali cement or the addition of supplementary cementitious materials may improve the mechanical properties and durability of wool fibre-reinforced cement-based composites. Accordingly, wool-reinforced cement-based composites are currently considered one of the most promising building materials in sustainable and eco-friendly engineering.

Nevertheless, future researches, aimed at improving the performance of sheep wool fibre reinforced cement-based composites, are needed. A special way to prepare the homogenous sheep wool fibres and modification of their surface will also be considered. Hybrid fibre reinforcement will be used to improve the mechanical properties and durability of the cement matrix composites. Investigations will be undertaken to increase the proportion of sheep wool fibres in cement-based composites.
